# Genome-Wide Identification of Basic Helix–Loop–Helix and NF-1 Motifs Underlying GR Binding Sites in Male Rat Hippocampus

**DOI:** 10.1210/en.2016-1929

**Published:** 2017-02-13

**Authors:** John R. Pooley, Ben P. Flynn, Lars Grøntved, Songjoon Baek, Michael J. Guertin, Yvonne M. Kershaw, Matthew T. Birnie, Annie Pellatt, Caroline A. Rivers, R. Louis Schiltz, Gordon L. Hager, Stafford L. Lightman, Becky L. Conway-Campbell

**Affiliations:** 1 Henry Wellcome Laboratories for Integrated Neuroscience and Endocrinology, University of Bristol, Bristol BS1 3NY, United Kingdom; 2 Department of Biochemistry and Molecular Biology, University of Southern Denmark, 5230 Odense, Denmark; 3 Laboratory for Receptor Biology and Gene Expression, National Cancer Institute, National Institutes of Health, Bethesda, Maryland 20892; 4 University of Virginia School of Medicine, Charlottesville, Virginia 22908

## Abstract

Glucocorticoids regulate hippocampal function in part by modulating gene expression through the glucocorticoid receptor (GR). GR binding is highly cell type specific, directed to accessible chromatin regions established during tissue differentiation. Distinct classes of GR binding sites are dependent on the activity of additional signal-activated transcription factors that prime chromatin toward context-specific organization. We hypothesized a stress context dependency for GR binding in hippocampus as a consequence of rapidly induced stress mediators priming chromatin accessibility. Using chromatin immunoprecipitation sequencing to interrogate GR binding, we found no effect of restraint stress context on GR binding, although analysis of sequences underlying GR binding sites revealed mechanistic detail for hippocampal GR function. We note enrichment of GR binding sites proximal to genes linked to structural and organizational roles, an absence of major tethering partners for GRs, and little or no evidence for binding at negative glucocorticoid response elements. A basic helix–loop–helix motif closely resembling a NeuroD1 or Olig2 binding site was found underlying a subset of GR binding sites and is proposed as a candidate lineage-determining transcription factor directing hippocampal chromatin access for GRs. Of our GR binding sites, 54% additionally contained half-sites for nuclear factor (NF)-1 that we propose as a collaborative or general transcription factor involved in hippocampal GR function. Our findings imply a dose-dependent and context-independent action of GRs in the hippocampus. Alterations in the expression or activity of NF-1/basic helix–loop–helix factors may play an as yet undetermined role in glucocorticoid-related disease susceptibility and outcome by altering GR access to hippocampal binding sites.

## Teaser

A stressful experience does not influence GR binding in the hippocampus relative to nonstressed controls. Insights into GR binding mechanisms include support via basic helix–loop–helix and NF-1 motifs.

The hypothalamic–pituitary–adrenal (HPA) axis responds to stress with a delayed secretion of glucocorticoids promoting structural and neurochemical changes within the brain that subserve the adaptive response to stress, but can also contribute to maladaptation resulting in disease states (1, 2). HPA axis abnormalities are well described as underlying features of a number of neuropsychiatric and neurodegenerative conditions, including major depression (3), posttraumatic stress disorder (4), social phobia (5), Huntington disease (6), and Alzheimer’s disease (7). These abnormalities may influence susceptibility to disease states (8) or contribute to the severity of clinical symptoms (9). Thus, understanding the mechanisms for glucocorticoid actions in the brain is an essential goal.

The glucocorticoid receptors (GRs) and mineralocorticoid receptors have genomic roles as ligand-dependent transcription factors that bind DNA and coordinate the regulation of gene expression networks in response to glucocorticoid (10–12). In this study, we focus on GRs, the dysfunction of which is thought to underlie altered negative feedback and HPA axis hyperactivity in major depression (13). Three forms of DNA binding have been described for GRs: direct DNA interaction at variants of the consensus glucocorticoid response element (GRE) motif 5′-ACAnnnTGT(T/C)CT-3′ (14, 15), direct DNA binding to negative GREs of variable sequence (16, 17), and indirect DNA binding through tethering of GRs to other transcription factors (18).

GR binding to GREs in hippocampus chromatin is incompletely understood, although chromatin organization reflects a restrictive environment for signal-activated transcription factors such as GR (19–21), and it accounts for transcription factors accessing only a small percentage of the total number of candidate DNA-binding motifs genome-wide (22). Access is “granted” by local remodeling and posttranslational modification of chromatin into accessible regions known as DNase I hypersensitive sites (DHSs) (23, 24). On average, there are tens of thousands of DHS-defined accessible regions in a cell type, generated and maintained by transcription factor–mediated recruitment of adenosine triphosphate–dependent remodeling proteins and histone-modifying enzymes that collectively modify nucleosome positioning and biochemical properties (25, 26). Transcription factor binding occurs almost exclusively at these regions of open and accessible chromatin (27, 28).

Crucially, the vast majority (up to 95%) of DHSs are preaccessible, that is, present prior to signal-dependent transcription factors such as GRs becoming activated (27, 29, 30). It is becoming increasingly evident that preaccessible DHSs arise due to remodeler recruitment via *trans*-acting factors that have been termed pioneers (31–35), licensing factors (33), initiators (36, 37), or lineage-determining transcription factors (LDTFs) (38–43). Despite the lack of naming consensus, it is generally accepted that these factors act to initiate chromatin access in a highly cell type–specific manner. Consequently, GR binding events and glucocorticoid actions are also highly cell type–specific delivered by the different accessible chromatin landscape for each cell type (21, 29, 37, 43, 44). As factors regulating cell type–specific chromatin access impact on enhancer selection, subtle changes in their expression and activity may be important in disease processes. Therefore, identification of factors influencing GR chromatin access in brain tissue may open new avenues of investigation for multiple disease states.

Glucocorticoid actions within the same cell, or body of cells, can also be context specific. Glucocorticoid-dependent facilitation of emotionally salient memory consolidation and retrieval requires activation of GRs in the amygdala and prefrontal cortex (PFC) but also noradrenergic signaling within the amygdala (45). Corticosterone enhancement of contextual fear memory consolidation in songbirds depends on the activation of hippocampal dopamine D1 receptors (46), whereas long-term potentiation is impaired in the hippocampus in a manner requiring glucocorticoids and the amygdala after stress (47). Interestingly, several cell line studies demonstrate an interplay with the accessible chromatin landscape as a mechanism for context-dependent glucocorticoid activity. GR activation contributes a *de novo* chromatin remodeling role, allowing coactivated estrogen receptors (ERs) to bind into genomic locations inaccessible to ERs when activated alone (48). To a lesser extent, ERs mediate chromatin access for coactivated GRs. Similar mechanisms likely explain the genome-wide redistribution of GR binding sites when GRs are coactivated with nuclear factor κB (49) or signal transducer and activator of transcription 3 (50). Effects on gene expression in the latter studies indicate a novel mechanism by which context can impact gene regulation within the same cell type. This mechanism may also account for differential transcription in neuroblastoma cells cotreated with dexamethasone and a *µ*-opioid receptor agonist compared with either ligand alone (51).

Continuing this line of investigation into animal tissues, signaling convergence is proposed to coordinate cross-talk between pathways necessary for stress-induced alterations in behavior and physiology (52). Many stress-induced alterations in brain signaling are rapid (milliseconds to seconds) compared with circulating glucocorticoids that peak with GR activity 30 minutes following restraint stress initiation (53, 54). This delay may allow rapid mediators of the stress response to prime the hippocampus for glucocorticoid signaling (52). We hypothesized that priming might involve reorganization of chromatin downstream of early stress mediators, redistributing GR binding and possibly governing some context-dependent physiological glucocorticoid actions. We examine GR occupancy genome-wide in the rat hippocampus for evidence of stress context redistribution of GR binding, finding that GRs actually occupy the same sites independently of context. Context was controlled by infusing corticosterone into adrenalectomized male rats while one group was subjected to a 30-minute restraint stress, whereas another group remained in the home cage [nonstressed control (NSC)]. Duplicate samples for each group additionally allowed for interrogation of candidate motifs underlying hippocampal GR binding sites. GR binding was discovered near genes linked to structural and organizational roles in the hippocampus. Mechanistic insights into GR function were derived from analysis of sequences underlying GR binding sites, including that tethering and binding at negative glucocorticoid response elements are not major contributors to GR–DNA interaction in the hippocampus. Binding is instead centered at GREs or their half-sites, supported by neighboring basic helix–loop–helix (bHLH) and nuclear factor (NF)-1 half-site motifs. NF-1 proteins are ubiquitous transcription factors characterized by the ability to act as both transcription and viral replication factors.

## Materials and Methods

### Animals and treatment

All animal procedures were performed in accordance with the revised UK Animals (Scientific Procedures) Act of 1986 and European Union Directive 2010/63/EU. Adult male Sprague-Dawley rats (225 to 250 g) had food and water *ad libidum* and were used to avoid complications with estrous, ER, or progesterone receptor activities that may influence GR function in a different context to the one tested. Rats received balanced anesthesia using veterinary isoflurane (Merial Animal Health, Woking, UK) prior to bilateral adrenalectomy and implantation of two right jugular venous polythene cannula for simultaneous blood sampling and infusion (55). Animals recovered for 5 days postsurgery on 15 µg/mL corticosterone in 0.9% saline drinking solution to maintain isotonic levels, which was replaced 12 hours prior to experiments with 0.9% saline.

All animals received infusions of corticosterone (0.75 mg/mL in the form of corticosterone 2-hydroxypropyl-*β*-cyclodextrin; Sigma-Aldrich, Gillingham, UK) dissolved in sterile 0.45% (w/v) NaCl. A New Era NE-1800 computer-driven infusion pump (World Precision Instruments, Aston, UK) delivered 1 mL/h for 30 minutes via one indwelling cannula to mimic the endogenous rise in corticosterone observed following restraint stress (53).

### Validation of experiment model

Animals were randomly assigned to NSC or stressed groups. Animals in the NSC group were left in their home cages whereas stressed group animals were restrained in Perspex cylinders for 30 minutes. Blood samples were taken via the second jugular cannula immediately before infusion (0 minutes), and at 10, 20, and 30 minutes into the infusion. Blood was collected into ice-cold heparinized tubes with 2500 kIU aprotinin (Trasylol; Bayer, Leverkusen, Germany).

### Tissue collection for chromatin immunoprecipitation sequencing experiments

After infusion, animals were euthanatized with 0.2 mL intravenous sodium pentobarbital via the second cannula. Whole hippocampus was dissected on ice for chromatin immunoprecipitation (ChIP), and PFC was removed and snap frozen in liquid nitrogen for quantitative reverse transcription polymerase chain reaction. Trunk blood was collected into ice-cold heparinized tubes for corticosterone measurement.

### GR ChIP sequencing

Hippocampi were fixed for ChIP and processed to chromatin in sodium dodecyl sulfate (SDS) lysis buffer [2% SDS, 10 mM EDTA, 50 mM Tris-HCl (pH 8.1)] as previously described (56). Chromatin was sheared with a Sonifier 450 (Branson Ultrasonics, Danbury, CT; 12× 10-second pulses at 10% output with 30 seconds on ice between pulses) and then cleared of cellular debris by centrifugation.

Each unique sample was composed of chromatin from eight hippocampi pooled together, divided, and processed as 15 separate 100-µg ChIP reactions. For each reaction, chromatin was diluted 1:10 in ChIP dilution buffer [167 mM NaCl, 16.7 mM Tris-HCl (pH 8.1), 1.1% Triton X-100, 1.2 mM EDTA, 0.01% SDS] supplemented with cOmplete protease inhibitor (Sigma). Chromatin (1%) input was collected and stored at −20°C until required. Reactions were immunoprecipitated overnight at 4°C with 2 µg anti-GR M-20 X (Santa Cruz Biotechnology, Dallas, TX), 4 µL anti-GR PA1-510A, and 4 µg anti-GR PA1-511A antiserum (both Thermo Fisher, Gloucester, UK). Antibody details are provided in Supplemental Table 1.

GR–DNA complexes collected onto protein A–conjugated Dynabeads (Invitrogen, Paisley, UK) were washed to remove nonspecific binding. Purified DNA was resuspended in water and samples for each group were concentrated into 30-µL volume with a Univapo vacuum concentrator (Transcriptomics Facility, University of Bristol). Two replicate ChIP and matched input samples per group underwent library preparation and sequencing on a GA2x using the Illumina platform at the National Cancer Institute Advanced Technology Center (Rockville, MD).

### NF-1 association with GR binding sites

ChIP performed as described above with sonication time reduced to 3× 10-second pulses at 10% output. Two micrograms of anti NF-1 H-300 (Santa Cruz Biotechnology) was used to ChIP 40 µg of chromatin. Equivalent amounts of nonimmune rabbit IgG (Santa Cruz Biotechnology) provided controls. Supplemental Table 2 lists quantitative polymerase chain reaction primers used to validate enrichments at specific sequences identified by ChIP sequencing (ChIP-seq).

### ACTH and corticosterone assays

Radioimmunoassay for plasma corticosterone and immunoradiometric assay for ACTH (Diasorin, Wokingham, UK) were performed as previously described (57) except for a 1:4 dilution of plasma in 0.9% saline for ACTH assays.

### PFC c-fos induction

Total RNA was extracted from rat PFC by the phenol–guanidine isothiocyanate–chloroform method as previously described (56) and further purified using an RNeasy kit with on-column DNase digestion according to the manufacturer’s instructions (Qiagen, Manchester, UK). One microgram of total RNA was converted to complementary DNA using an AMV first-strand synthesis kit (Invitrogen) and *c-fos* expression quantified by quantitative reverse transcription polymerase chain reaction using a TaqMan primer-probe assay (no. Rn02396759_m1; Applied Biosystems, Warrington, UK) on an ABI Prism 7500 detection system (Applied Biosystems). Data were normalized to the *β*-*actin* housekeeping gene (assay ID 4352931E; Applied Biosystems), and relative expression was analyzed by the ΔΔCt method.

### Statistical analysis

Hormone levels in blood samples taken over the time course were analyzed using repeated measures analysis of variance (ANOVA) with Petersen replacements (58) for missing values. Significant main effects were reported with Greenhouse–Geisser correction for unequal variance when necessary. *Post hoc* testing used a Wilcoxon signed-rank test between time points or a Mann–Whitney *U* test between groups, with each Bonferroni adjusted for multiple comparisons. End-point corticosterone levels and PFC *c-fos* expression were both compared using two-way ANOVA.

### ChIP-seq data analysis

Alignment to the rn5 reference genome was performed using Bowtie2. Tags were extended to 150 bp in their 3′ direction, and duplicated tags were removed, eliminating polymerase chain reaction bias. Tag density was normalized to the appropriate input controls, and simple repeats were masked for replicate concordant peak calling using an in-house algorithm Hotspot (27, 34, 59). Significantly enriched genomic regions common to both replicates were ranked by tag density, and the top 20% in each group were carried through to additional analysis. GR peaks were visualized in the University of California Santa Cruz Genome Browser (60) with groups normalized to sequencing depth (number of tags per 10 million total reads). DAVID gene ontology was used to explore the functional relationship of likely GR targets (61).

HTSeq (62) counted raw tags at GR binding sites before DESeq (63) compared peak intensities statistically. After adjusting for the number of comparisons, *P* values of <0.05 were taken as significant. HOMER Analysis Suite v4.7 (39) annotated GR peaks to their nearest transcription start site. Promoters were defined as −5 kb to +100 bp to report genomic locations of GR binding sites. GR binding sites were arranged according to maximum tag density before HOMER counted raw tag density 3 kb either side of the peak center. Data were exported to MeV version 4-8-1 (Dana–Farber Cancer Institute, Boston, MA) for heat map generation.

GR binding sites were analyzed for known and *de novo* motifs using HOMER. Repeat sequences were masked and motifs were optimized around 6, 8, 10, 12, 14, and 16 bp. Motif counting data using known motif files and histograms indicating motif positions relative to the peak center were produced in HOMER. Motif files matching the three variants of the consensus sequence for negative GREs, 5′-CTCC(N)_0-2_GGAGA-3′ (17), were created using motif editor allowing only exact matches to the negative GRE (nGRE) consensus.

GEO accession GSE46047 provided DHS sequencing (DHS-seq) and GR ChIP-seq peak annotations for adrenalectomized mouse liver. Assay for transposase-accessible chromatin sequencing (ATAC-seq) data for neuronal subtypes were available under GEO accession GSE63137. DHS sequencing (DHS-seq) data for human primary astrocytic cells (HA-h, HA-c, HA-sc) and for the human neuronal SK-N-MC cell line were obtained from the ENCODE database. GEO accession GSE61236 provided DHS-seq and GR ChIP-seq peak annotations for 3134 cells with and without corticosterone treatment.

## Results

### Validation of experimental design

This study was designed to investigate the potential for context-dependent alterations in GR binding in the rat hippocampus. Robust validation of the experimental design was required to ensure well-matched corticosterone levels while additionally maintaining basal HPA axis activity in one group but activating the central stress pathways in the second group.

**Figure 1.**
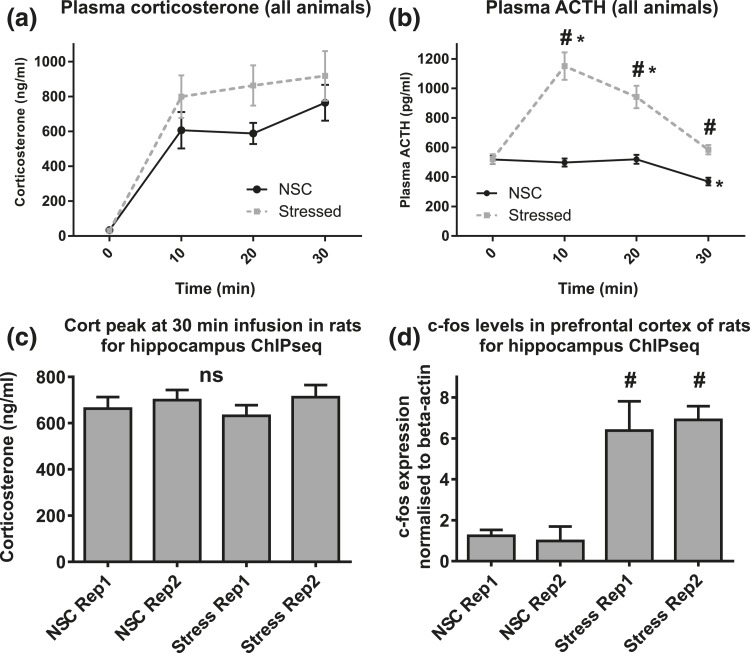
Validation of experimental design. (a) Plasma corticosterone measured prior to the start of the infusion (0 minutes) and at 10, 20, and 30 minutes thereafter. Blood samples were drawn by hand through indwelling cannulas. Corticosterone rises after the start of the infusion and does not differ between NSC (black circles, n = 30 to 31) and stressed (gray squares, n = 17 to 20) groups. (b) Plasma ACTH levels rose in stressed group rats, in line with activation of the central stress response. ^#^*P* < 0.0001 (between groups); *P < 0.005 (different from 0 minutes). n = minimum of 30 (NSC) or 17 (stressed). (c) Plasma corticosterone in trunk blood for animals taken for ChIP-seq. All animals received similar corticosterone infusions regardless of group or replicate (one-way ANOVA, n = 8 per replicate). (d) PFC *c-fos* expression is significantly different between groups. The two stressed group replicates have higher *c-fos* levels compared with either NSC group. ^#^*P* < 0.0001 (compared with NSC replicates), n = 8. NSC replicates were not different from each other, and stressed replicates were not different from each other. Means ± standard error of the mean are shown.

Circulating corticosterone levels increased in both groups during the infusion [significant effect of time; *F*(2.33,137.6) = 54.1, *P* < 0.0001]. There was no significant interaction of time × group [*F*(2.33,137.6) = 0.51, *P* = 0.629] and no effect of group [*F*(1,59) = 2.58, *P* = 0.113], indicating that corticosterone levels were consistent across groups [Fig. 1(a)].

Plasma ACTH showed significant main effects of time [*F*(2.21,130.1) = 35.0, *P* < 0.0001)] and of group [*F*(1,59) = 42.2, *P* < 0.0001]. An interaction (time × group) *F*(2.21,130.1) = 9.75, *P* < 0.0001 indicated that ACTH levels changed in both groups but with different patterns [Fig. 1(b)]. *Post hoc* testing showed that ACTH in stressed rats increased significantly 10 minutes following commencement of restraint (*P* = 0.001), demonstrating activation of the central stress response. ACTH then returned to prestress levels by 30 minutes (*P* = 0.096). In the NSC group there was a small but significant decrease in ACTH at 30 minutes (*P* < 0.0001), but levels were otherwise stable. Importantly, both groups began with comparable ACTH levels (*P* = 0.626), but the stressed group had higher ACTH at all other time points (each *P* < 0.0001).

For the eight animals pooled per replicate for ChIP-seq, there was no difference in trunk blood corticosterone levels between groups [*F*(1,28) = 0.038, *P* = 0.847] or between replicates [*F*(1,28) = 1.481, *P* = 0.234] [Fig. 1(c)]. PFC *c-fos* expression showed a significant effect of group [*F*(1,28) = 318.0, *P* < 0.0001] but no effect of replicate [*F*(1,28) = 0.193, *P* = 0.663] [Fig. 1(d)], indicating activation of the central stress response (64) only in the expected group.

### ChIP-seq detects previously known and novel GR binding sites in rat hippocampus

**Figure 2.**
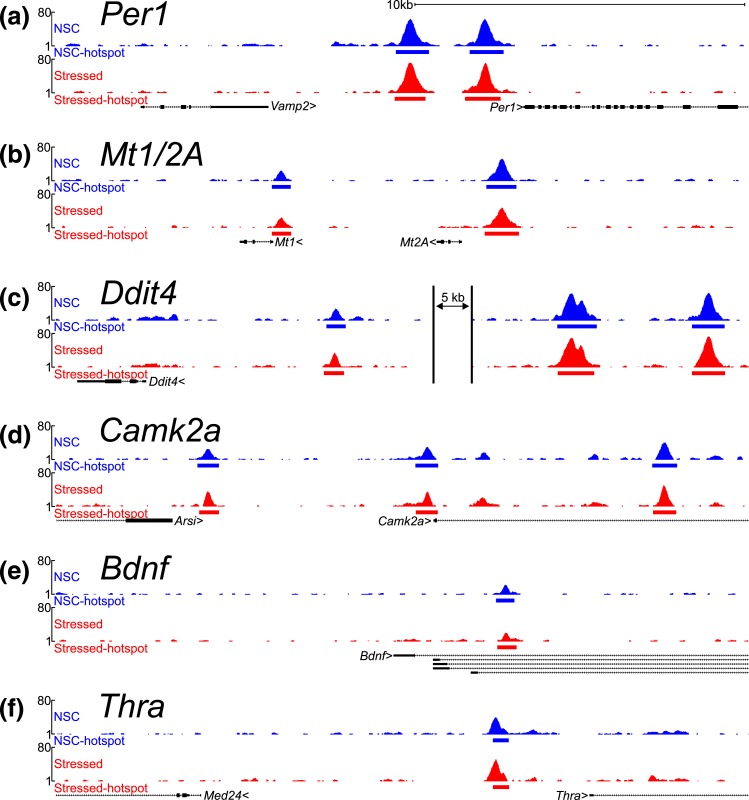
GR ChIP-seq in rat hippocampus reveals known and unknown GR binding sites. (a–f) Browser tracks showing regions of interest in the genome. Tag density profiles are shown for each group indicating regions called as GR-bound peaks. Known genes (RefSeq) are indicated along with the direction of the coding strand (chevron). (a and b) Promoter region for the clock gene period 1 (*Per1*) known to contain binding sites for GRs in rat hippocampus (56), and the promoter region of metal ion chelator metallothionein-2a (108). (c) The upstream region of the Ddit4 gene also known to contain GR binding sites. The presence of these known sites within the peak list highlights the success of the experimental protocol. (d–f) Novel GR binding sites in the hippocampus. Several locations flanking the calcium-calmodulin–dependent kinase 2a (*Camk2a*) gene and the promoter of the thyroid hormone receptor *α* (*Thra*) gene bind GRs. A weaker intronic binding site is also observed in the *Bdnf* gene.

Formaldehyde-fixed nuclei from whole hippocampus (eight rats per replicate) underwent ChIP-seq for GR. Replicate concordant peaks were ranked by their maximum tag density, and the strongest 20% (highest number of tags) for each group were selected for downstream analysis. Examples of the 7298 total genomic regions containing GR binding sites in stressed and NSC rat hippocampi are shown in Fig. 2. Binding sites previously described in hippocampus [Fig. 2(a–c)] as well as novel target sites for GRs [Fig. 2(d–f)] were observed. Sequencing files are available on National Center for Biotechnology Information GEO DataSets under accession number GSE94008.

### No apparent redistribution of GR binding sites in the context of restraint stress

**Figure 3.**
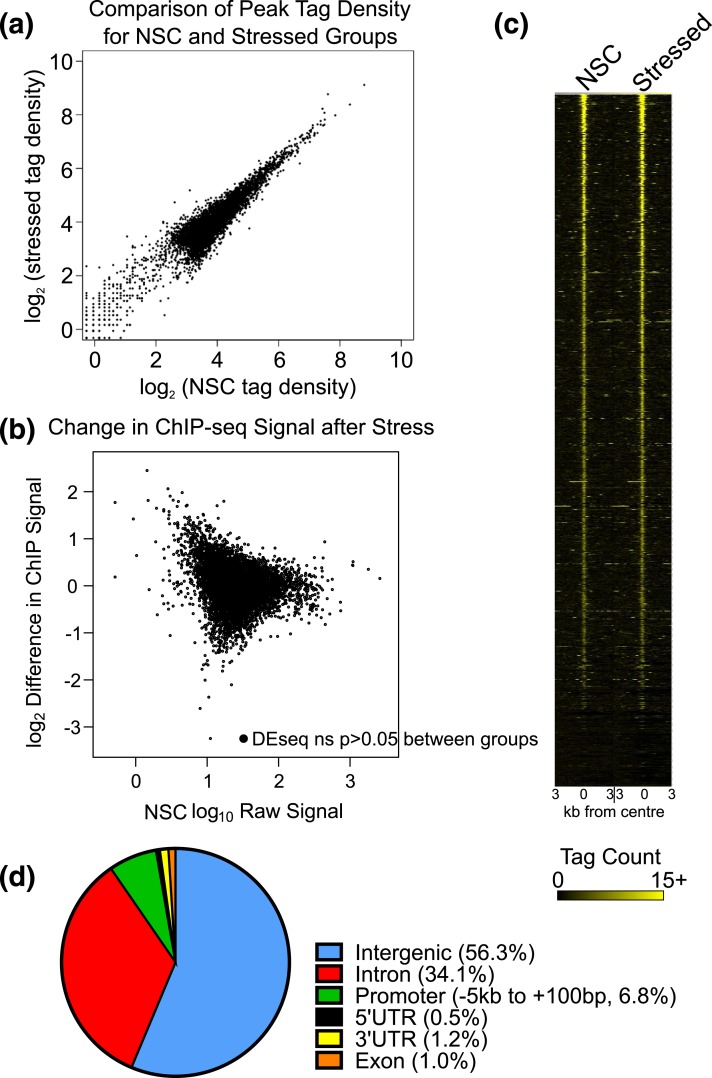
(a) Scatter plot of the log_2_ of peak intensity at the same chromosome position in NSC and stressed groups. (b) Log ratio (M) versus mean average (A) plot for NSC and stressed peaks. Peaks significantly different between groups were set to display as red dots. There are no peaks significantly different between groups by DESeq. (c) Heat map plotting tag density over the center of the peak (±3 kb) supports the view that GR binding is not significantly different between groups. Peaks are ranked according to their tag density. (d) Distribution of hippocampus GR binding sites across the genome annotated according to nearest transcription start site. Most binding events occurred in intergenic or intronic regions with comparatively little occurring at promoters or within coding regions. UTR, untranslated region.

To test for changes in hippocampal GR binding genome wide related to the context of stress exposure, the tag density between NSC and stressed groups were compared at all peak chromosome positions. Figure 3(a) indicates that peaks have highly similar tag densities between groups (Pearson correlation 0.920, *P* < 0.0001). DESeq analysis using both replicates from each group revealed that no peaks had significantly different tag densities between groups [log ratio (M) versus mean average (A) plot, Fig. 3(b)]. A heat map displaying all the chromosome positions ranked by peak tag density [Fig. 3(c)] enabled clear visualization of the lack of differences between the two groups. All nonduplicated genomic positions were therefore pooled for further analysis (Supplemental Table 3).

### Distribution of GR binding sites

GR binding site distribution was examined by annotating chromosomal position relative to the nearest gene transcription start site. As has been observed previously for GRs in the rat hippocampus (65), most GR binding occurred in intergenic regions or within introns [Fig. 3(d)]. Fewer binding events were observed in promoters, 3′ or 5′ untranslated regions or exons.

To uncover hippocampal genes that may be influenced by activated GRs, we isolated 3165 binding sites located inside a gene body (intron, exon, or untranslated region) or the extended promoter region (upstream to −5 kb). Gene ontology analysis revealed significant enrichments for 480 biological function categories (Supplemental Table 4) and 146 significantly enriched molecular functional categories (Supplemental Table 5). These data suggest that glucocorticoid hormones, via GRs, influence the regulation of genes disproportionally linked to the molecular processes of ion binding, phosphorylation, cytoskeleton, and voltage-gated channel activity. Functional annotations include regulation of development, morphogenesis, and organization of cells of the central nervous system and their projections in addition to the transmission of electrical information between cells.

### Identification of candidate *trans*-acting factors contributing to GR binding in rat hippocampus

In the absence of stress context–dependent GR binding in rat hippocampus, we postulated that stress-activated transcription factors were not involved in either redefining the hippocampal accessible chromatin landscape in this time frame or acting as a major GR tethering partner. DNA sequences underlying GR binding sites in both the present study and that of Polman *et al.* (65) were subjected to *de novo* motif analysis to interrogate which DNA binding motifs were enriched at hippocampal GR binding sites.

**Figure 4.**
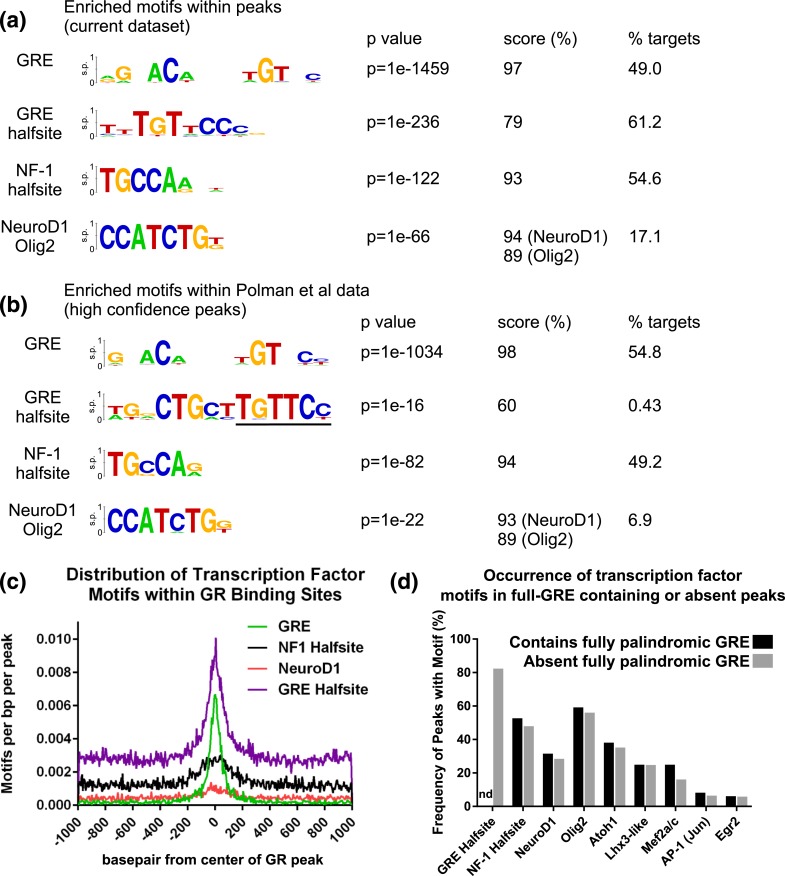
Motif analysis identifies candidate binding partners for GRs in rat hippocampus. (a) *De novo* motif analysis of DNA sequences underlying GR peaks in rat hippocampus returns motifs that strongly resemble a palindromic GRE, NF-1 half-site, and a basic helix–loop–helix transcription factor most likely NeuroD1 or Olig2. The *P* value shows significance level for the enrichment of the motif indicated. The score indicates the similarity of the identified motif to the known motifs for the factor indicated, with higher percentages equivalent to greater confidence in an accurate match. The percentage of peaks containing motifs that match this model is also indicated (percentage targets). s.p., specific probability. (b) GR binding sites in rat hippocampus identified with high confidence by Polman *et al.* (65) were additionally examined by *de novo* motif discovery using the same settings as above. A motif matching a GRE half-site (underline) was more degenerate and poorly represented within peaks overall. (c) Motifs for the factors identified are located toward the center of the GR binding site. (d) The frequency of discovered motifs was separately determined for GR peaks containing a fully palindromic GRE, and for the remaining GR peaks that did not contain a GRE palindrome. By default, all GR peaks containing a full GRE also contained a GRE half-site, and so this was not determined (nd) for the full GRE containing population. Most peaks absent a full GRE contained a GRE half-site instead.

The most significant *de* *novo* motif discovery for our data set was similar to the GRE (similarity score 97%) and was present in 49% of all GR peaks. A motif resembling a GRE half-site (score 73%, 61.2% of all peaks) was also a significant finding in our data set. A motif closely resembling the NF-1 half-site (score 93%, 54.6% of all peaks) and a motif matching a bHLH transcription factor were additionally enriched (17.1% of all peaks). The latter motif best matched the consensus DNA binding site for the transcription factor NeuroD1 (score 94%), as well as displaying a high degree of similarity to the consensus motif of the related Olig2 (score 89%) [Fig. 4(a)]. Similar motifs were obtained from the high confidence peak list of Polman *et al.* (65) when reanalyzed using the same parameters as our own data [Fig. 4(b)]. A principal difference in the reanalyzed data set was the GRE half-site motif, which was weakly represented and degenerate relative to that discovered in our GR binding sites. Non–GRE motifs occurring in GR binding sites were located toward the center of GR binding [Fig. 4(c)] but displayed a broader distribution from the peak center than did GREs, suggesting more positional flexibility.

A common tethering factor for GR in hippocampus would be expected to present a distinct motif. Of our total identified GR binding sites, 3997 did not contain a fully palindromic GRE and were separated from those that did for additional analysis. *De novo* motif analysis of these two subsets of peaks—group 1 containing a full GRE and group 2 absent a full GRE—produced additional motifs with moderate-high confidence matches to known transcription factor binding sites (Supplemental Fig. 1). Nonetheless, motif frequency analysis for peaks in these two groups produced highly similar findings [Fig. 4(d)]. Of peaks absent a full GRE, 81.6% contained GRE half-sites that likely supported GR binding. Overall, 89.9% of all GR binding sites contained some form of GRE. Further analysis of 735 GR binding sites that contained neither a full GRE nor a GRE half-site did not yield any additional consensus motifs.

**Figure 5.**
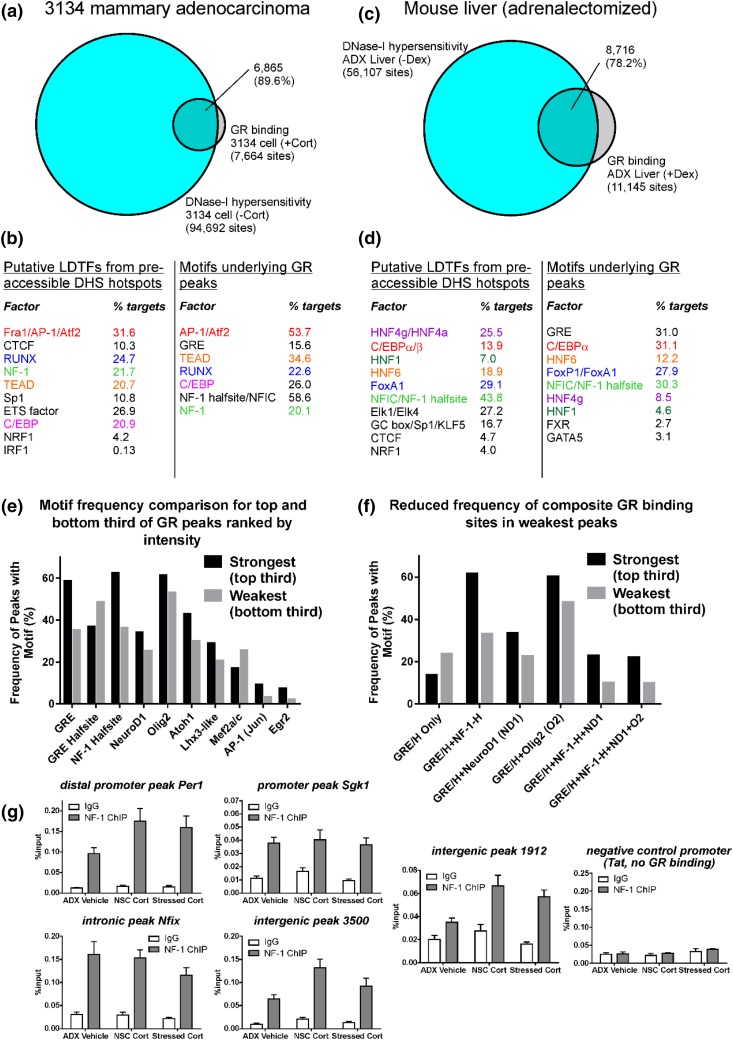
Overlap of GR binding sites (with dexamethasone or corticosterone) with preaccessible chromatin defined by DNase I hypersensitivity (without dexamethasone or corticosterone) in the (a) 3134 mammary carcinoma cell line, and (c) in adrenalectomized mouse liver. Redrawn from data in Stavreva *et al.* (67) and Grøntved *et al.* (37), respectively. *De novo* motif discovery for DHS peaks and GR binding sites in (b) 3134 cells, or (d) mouse liver, produces many of the same motifs. Motifs are arranged in order of significance with *P* values for DHS motifs between 1e-7713 and 1e-42, and *P* values for GR binding site motifs between 1e-2918 and 1e-53. All motifs indicated have a confidence score of >80%. (e) GR binding enrichment was determined by ordering peaks according to tag density and counting motifs in the top and bottom third, respectively. Weaker peaks have fewer occurrences of transcription factor motifs. GREs, NF-1 half-sites, NeuroD1, Olig2, Atoh1, Lhx3-like, AP-1, and Egr2 motifs particularly are less well represented in weaker GR binding sites. Conversely, GRE half-sites and myocyte enhancer factor 2 motifs are better represented in weaker binding sites. (f) Composite motifs containing GREs, NF-1, and NeuroD1/Olig2 are more common in stronger peaks whereas weaker binding sites are more likely to contain isolated GRE sequences. GRE/H, full GRE or GRE half-site; NF-1-H, NF-1 half-site; ND1, NeuroD1; O2, Olig2. (g) ChIP assay showing the presence of NF-1 at four out of five selected GR binding sites in rat hippocampus is not corticosterone/GR-dependent. Intergenic peak 1912 may be a *de novo* site requiring GR to recruit NF-1. The liver specific *Tat* gene promoter does not recruit GRs in this tissue and is shown as a negative control for NF-1 binding. Means ± standard error of the mean are shown; n = minimum of 7 for all sites except the *Tat*-negative control, where n = a minimum of 3.

We next explored whether there might be functional significance associated with the motifs overrepresented within the entire set of hippocampal GR binding sites. It is now quite well established that the accessible chromatin landscape of each different cell type depends on transcription factors classically referred to as pioneers (35), and more recently as initiators (36). The behavior of such factors shares similarities with LDTFs proposed by others (39). As both LDTFs and pioneers define accessible chromatin, their DNA recognition sequences commonly occur within accessible sites. Because most GR DNA binding occurs in preaccessible sites (27), motif analysis of GR binding sites also recovers pioneers/LDTFs (37, 66). We amalgamated these concepts and present this relationship by reanalyzing published GR binding (ChIP-seq) and accessible chromatin (DHS-seq) data from cell line 3134 and mouse liver (37, 67). As most GR binding sites are within preaccessible chromatin [Fig. 5(a) and 5(c)] defined by LDTFs, many GR binding sites also possess motifs for putative LDTFs [Fig. 5(b) and 5(d)]. Taken together, these data imply that NF-1 half-sites and NeuroD1/Olig2 motifs underlying hippocampus GR binding sites might reflect LDTFs for the multiple cell types comprising the hippocampal formation.

We therefore tested whether there was any evidence for greater GR enrichment at sites containing NF-1/bHLH motifs, as was reported for GRs in liver at binding sites containing CCAAT/enhancer-binding protein (37). Consistent with the dynamic assisted loading mechanism proposed to account for this observation in liver (36, 68), NF-1/bHLH motifs were more frequently associated with highly enriched (strong) GR binding sites vs weaker sites [Fig. 5(e)]. This effect was also quite marked for AP-1 and Egr2, although these motifs occurred in considerably fewer peaks. We next examined the frequency of composite sites—NF-1 half-sites and NeuroD1/Olig2 motifs with GREs—in strong and weak GR binding sites [Fig. 5(f)]. Fully palindromic GREs or GRE half-sites occurred alone more commonly in weak peaks compared with strong, whereas strong peaks more frequently contained combinations of GREs with the additional motifs.

Most GR binding sites are within preaccessible chromatin defined by LDTFs [Fig. 5(a–d)] and accessible prior to hormone treatment. Thus, we might expect that recruitment of candidate LDTFs NF-1 and NeuroD1/Olig2 would not be hormone-dependent at a large majority of sites. Choosing to focus on NF-1 for which a suitable antibody was available, ChIP assay revealed that the presence of NF-1 was not dependent on corticosterone or GR activity at four out of five GR binding sites [Fig. 5(g)].

As hippocampus contains many different cell types, we have investigated which cell types are most likely responsible for each motif obtained. Separate *de novo* motif discovery on ENCODE DHS-seq data for three populations of human primary astrocytes (hippocampus, cortex, and spinal cord) revealed candidate LDTFs mediating accessible chromatin in astrocytes. We noted that NF-1 half-sites were present in all three subtypes but NeuroD1/Olig2 motifs were absent (Table 1). To our knowledge, similar data for oligodendrocytes or microglia are not yet available. ATAC-seq data for purified populations of excitatory pyramidal neurons, vasoactive intestinal peptide–expressing interneurons, and parvalbumin-expressing interneurons from mouse neocortex have been published (69). ATAC-seq provides similar information to DHS-seq, and *de novo* motif analysis indicated that neuronal subtypes shared several candidate LDTFs whereas others were unique to neuron type (Table 1). A NeuroD1/Olig2 motif was uniquely detected in excitatory pyramidal cell accessible chromatin in addition to 17.1% of hippocampus GR binding sites. Variant bHLH motifs were detected in interneurons but more closely matched other bHLH transcription factors whereas Lhx2 was unique to vasoactive intestinal peptide–expressing interneurons. NF-1 half-sites were again detected in all primary neuronal subtypes, appearing in a greater percentage of accessible chromatin sites compared with astrocytes.

**Table 1.** ***De Novo* Motif Discoveries in Accessible Chromatin From Astrocytic and Neuronal Primary Cells and From the SK-N-MC Neuronal Cell Line for Comparison**

**Motif Identified (Score >85%)**	**Astrocytes**			**Primary Neurons**			**Neuronal Cell Line** **SK-N-MC**
	**HA-h**	**HA-c**	**HA-sc**	**Excitatory Pyramidal**	**VIP Interneuron**	**PV Interneuron**	
AP-1	1e-757 (26.6%)	1e-820 (25.3%)	1e-1035 (29.9%)	1e-301 (20.2%)	1e-130 (18.4%)	1e-160 (18.1%)	1e-65 (10.2%)
CTCF	1e-327 (5.7%)	1e-641 (9.2%)	1e-443 (6.9%)	1e-271 (7.0%)	1e-291 (9.6%)	1e-343 (11.3%)	1e-738 (12.2%)
RUNX	1e-196 (19.1%)	1e-257 (25.0%)	1e-180 (18.3%)				1e-122 (9.7%)
NF-1	1e-186 (15.0%)	1e-175 (18.1%)					1e-182 (6.5%)
TEAD	1e-248 (20.4%)	1e-151 (16.4%)	1e-152 (18.3%)				
NF-1 half-site/NFIC	1e-104 (16.5%)	1e-104 (24.9%)	1e-81 (17.5%)	1e-150 (47.8%)	1e-87 (47.6%)	1e-41 (60.4%)	
REST	1e-59 (0.4%)	1e-37 (0.7%)	1e-38 (0.4%)				1e-49 (0.3%)
Rfx family	1e-92 (2.5%)	1e-69 (4.4%)		1e-105 (3.4%)	1e-27 (4.3%)		1e-43 (1.8%)
Smad3		1e-24 (18.0%)					
Atf7		1e-83 (10.1%)	1e-71 (19.9%)				
Egr2				1e-322 (27.5%)			
bHLH NeuroD1/Olig2				1e-230 (21.9%)			1e-53 (7.5%)*^a^*
Mef2a/Mef2c				1e-148 (33.7%)	1e-73 (39.4%)*^b^*	1e-184 (24.0%)	
NFIL3				1e-62 (9.1%)		1e-34 (10.4%)	
bHLH Ap4/Ascl1/Tcf12					1e-192 (47.9%)	1e-107 (38.3%)	
Lhx2					1e-124 (41.2%)		
RORγt/RORA						1e-62 (7.0%)	
ETS-like (Fli1)							1e-229 (20.3%)
C/EBP family							1e-92 (26.3%)
GC-box: Klf/Sp1 family							1e-53 (7.0%)

Motifs matching the indicated transcription factors with >85% scores indicate high confidence in accurate identifications. The *P* values indicate significance for the motif enrichment within the data set, and in parentheses the percentage of total sites containing the indicated motif. For several factors multiple candidate identifications are made as the motif closely resembles each, or a family is indicated. SK-N-MC cells once described as a neuroblastoma cell line share motifs with both astrocytes and neurons in addition to unique motifs not found in primary cells. bHLH transcription factors are categorized according to motif. One group contains a motif (CAgATGG) that closely matches NeuroD1/Olig2 type transcription factors (lowercase indicates a more flexible base). Another contains motif CAgcTG, which better matches Ap4- and Ascl1-type factors.

Abbreviations: C/EBP, CCAAT/enhancer binding protein; HA-c, human astrocyte of the cortex; HA-h, human astrocyte of the hippocampus; HA-sc, human astrocyte of the spinal cord; PV, parvalbumin; VIP, vasoactive intestinal peptide.

^a^ A motif similar to the NeuroD1/Olig2 bHLH group is found in SK-N-MC cells but identification as TAL1 (score 89%) was preferred to Olig2 (86%) (column heading, SK-N-MC).

^b^ An Mef2-type motif is present (column heading, VIP neurons), but the motif does not reach the >85% confidence level for identification as Mef2a/c.

### Little support for GR binding at putative negative GREs

Previous reports suggest GR binding at nGRE motifs within genes related to the HPA axis and circadian regulation (17, 70). The hippocampus is well associated with HPA axis regulation (71), and we identified 126 hippocampal GR peaks containing nGRE sequences. Analysis revealed that all but six sites also contained palindromic or half-site GRE sequences that were on average closer to the peak center than the nGREs (Supplemental Fig. 2). Consequently, binding of GRs to most sites containing nGREs appeared more likely mediated through conventional GRE-like motifs. In the six sites where no obvious GRE or half-site could be observed, the nGRE was not central to the peak in five, suggesting that another factor was responsible for GR binding. The remaining site additionally contained the sequence 5′-GTCACAnnnAGTCCT-3′, which, although a degenerate motif, could conceivably bind GR.

## Discussion

### Absence of stress context in GR binding

In contrast to studies in cell lines where differential activation of signal-dependent transcription factors causes a marked redistribution of GR binding (48–50), we did not detect any stress-dependent redistribution of GR binding in the rat hippocampus. Various possible explanations might explain this outcome in a brain region where multiple signaling systems are influenced rapidly by the acute stress response. One possibility may be the inherent differences between cell lines and tissue. There is also considerable cellular heterogeneity in the hippocampus. It is therefore possible that a combination of these two factors—*in vivo* tissue comprising a mixed cell population—contributes to the inability to detect context-dependent GR binding alterations. As a consequence, potentially strong effects in weakly represented cell types might be lost in the average population. Alternatively, there is some evidence to support at least one major context-dependent glucocorticoid action in the hippocampus being dependent on peripheral adrenergic signaling (72), removed here by adrenalectomy.

Nevertheless, our data strongly indicate that in this experimental protocol acute restraint stress does not cause a redistribution of hippocampal GR binding sites. This supports a conclusion that the GR binding profile in the hippocampus is dose-dependent and not further modulated by concomitant activation of central stress-induced signaling pathways. Therefore, it seems more likely that any differential outcome in the behavioral and/or transcriptional response to stress is mediated by post-GR binding mechanisms. In support of this, transcription factors induced by stress, including Fos (73–75), CREB downstream of *β*-adrenergic receptor and CRH receptor signaling (76–79), and factors downstream of extracellular signal-regulated kinase and c-Jun N-terminal kinase cascades (*e.g.*, Elk1, Egr1, AP-1) (79–81), have been reported to exhibit a delayed onset. Therefore, it is conceivable that stress-associated changes might modify the chromatin landscape for subsequent stress exposure, thus providing a mechanism for how stress history alters the transcriptional response to glucocorticoid challenge (82).

### GR binding sites may confer regulatory influence to genes directing the production and organization of neural cells and their projections

GR binding sites were distributed around the genome comparable to many transcription factors, and as previously reported for hippocampal GRs (65). We performed gene ontology analysis to identify any functional significance that could be attributed to our GR binding sites. In addition to pathways consistent with neural GR actions, we found an enrichment of genes associated with the development and organization of cells of the nervous system. Related enriched categories included cell adhesion, cell projection organization, neuron development and differentiation, axonogenesis and synaptogenesis, and neuron projection development. Several structurally related genes identified (*e.g.*, *Bdnf*, *Egfr*, *Fgf2*, *Mertk*, and *Cyfip1*) are regulated by glucocorticoids at the mRNA level in hippocampus (82). Interestingly, some of these are additionally known to be dysregulated in the hippocampus of human patients with neuropsychiatric disorders (*e.g.*, *Bdnf*, *Fgf2*) (83, 84). Stress maladaptation in animal models produces defects in dendritic spine morphology and numbers, neurogenesis, glial cell density, and synapse formation (85–87). In stress-related neuropsychiatric disease, postmortem studies so far suggest similar findings (88–90), but may benefit from further study.

### Mechanisms for hippocampal GR action

We found little evidence of nGRE usage whereas 89.9% of hippocampal GR binding sites contained some form of GRE (full palindrome or half-sites). GR tethered to another factor produces a narrow motif distribution for that factor around the binding center (50). NF-1 and NeuroD1 motifs were more broadly distributed from peak centers than were GREs, and there is no existing evidence for protein–protein interactions between GRs and NF-1, NeuroD1, or Olig2. These motifs were found alongside GREs or GRE half-sites rather than instead of them. Taken together, these data indicate that tethering of GRs to DNA-bound transcription factors is not a major mechanism contributing to GR activity in the hippocampus as it is for some cell types (50). Similar conclusions were reached by others (65), although we differ in our assessment of palindromic GRE usage. Consistent with previous work in tissue (37), we detected multiple sites supported by GRE half-sites whereas the aforementioned work (65) did not. This might relate to increased sequencing depth in our study. Weaker sites, supported by GR half-sites, could potentially have been missed due to less sequencing depth reported in the earlier study (65). Indeed, our motif reanalysis of these previously reported peaks identified a weak match to a degenerate half-site [Fig. 4(b)]. However, as 735 hippocampus GR binding sites in the present work contain neither full nor half GREs, we cannot rule out tethering as a minor contributor to GR function, but no further consensus motifs were enriched in this population.

### Functional significance of transcription factor motifs underlying GR binding sites

DNA sequences for hippocampal GR peaks contained NF-1 half-sites and a motif reflecting a bHLH transcription factor binding site. The latter was identified computationally as NeuroD1 but could reflect Olig2 or a variety of other similar transcription factors such as Ascl1/Mash1, Olig1, Atoh1, Neurog2, and Hes (91, 92). Our motif discovery shows a surprising lack of agreement with candidate motifs identified in the only other published hippocampal GR ChIP-seq data set (65), likely due to differences in analysis. Accordingly, results from our HOMER reanalysis of the previously published hippocampal GR binding sites revealed the same motifs as our own data set. Similar analyses in cells and tissues reveal motifs for transcription factors involved in establishing accessible chromatin during differentiation that are described as pioneers or LDTFs (34, 37, 44, 66) (Fig. 5). Factors that bind to the motifs underlying hippocampal GR peaks might therefore be expected to have clear links to neural cell differentiation and brain development. Indeed, *NeuroD1*-deficient mice fail to develop a hippocampal granule cell layer (93). Survival and differentiation of adult born neurons from neural stem cells also require NeuroD1 (94), and low *NeuroD1* expression reduces neurogenesis (95). Knockout of related *Neurog2* also profoundly affects development of the dentate gyrus (96) whereas double-knockout of *Olig1/2* results in total loss of oligodendrocytes throughout the brain (97). Nfib and Nfix particularly contribute to hippocampus formation development (98, 99), and mice heterozygous for *Nfix* have highly abnormal hippocampal structure (100).

We show a broader tolerance of NF-1/NeuroD1/Olig2 motif positioning that need not be at the peak of GR binding, and that stronger GR binding sites contain more motifs for NF-1/NeuroD1/Olig2. Sample heterogeneity appreciably complicates interpretation of the presence of more motifs in stronger peaks, but the presence of NF-1 does not depend on GR activity at several sites [Fig. 5(g)], consistent with a role for NF-1 in defining preaccessible chromatin. As is the case for other pioneers, we hypothesize that most sites would load NF-1 prior to hormone and promote GR binding (44), although at a small percentage of sites, NF-1 would require GRs to access DNA (36) [*e.g.*, intergenic peak 1912, Fig. 5(g)]. Taken together, our findings suggest that NF-1 and NeuroD1/Olig2 support GR binding as candidate LDTFs. An architectural role for NF-1 in GR binding has been proposed by others (101), and similar mechanisms may occur in the hippocampus. Nonetheless, we offer only indirect evidence for NF-1 and NeuroD1/Olig2 involvement in establishing the accessible chromatin landscape in hippocampal cell types.

Heterogeneity makes the identification of cell types contributing to the NF-1 and NeuroD1/Olig2 motifs observed challenging. A sizable portion of cells in the present study were likely glial, consistent with a higher proportion of glial cells compared with neurons (1.6- to 3.0-fold) in adult rat hippocampus (102). Neurons and glia are further and extensively subdivided into a myriad of different cell types for which there is little or no information regarding abundance. We exploited published ATAC-seq and DHS-seq data sets reporting accessible chromatin for neurons and astrocytes. Although data from these methods are not absolutely concordant, both approaches identify accessible chromatin and show good overlap when applied to the same cell type (103).

All three types of neurons examined produced AP-1 and a bHLH factor motif in accessible chromatin. Only excitatory neurons isolated by *Camk2a*-driven green fluorescent protein expression (69) produced NeuroD1/Olig2 as a candidate LDTF. This motif was not present in any of the three types of astrocytes examined, suggesting that NeuroD1 comes from excitatory neurons. However, as other cell types could not be studied (*e.g.*, oligodendrocytes, microglia), this determination is tentative. Indeed, Olig2, a predominant transcription factor determining oligodendrocyte fate (97), would be expected to bind this motif.

Notably, NF-1 half-sites detected at 54.6% of hippocampus GR binding sites were also present at 47% to 60% of neuronal accessible chromatin sites, and at 16% to 25% of accessible chromatin sites in astrocytes (Table 1). This motif is also featured in the accessible chromatin of the mouse liver (Fig. 5), but it is not present in the accessible chromatin of neuronal SK-N-MC cells (Table 1), and it does not underlie GR binding sites in macrophages (66). Taken together, these results suggest some degree of cell specificity in the role of NF-1 in chromatin accessibility and GR binding. Therefore, despite sharing some LDTF characteristics, NF-1 may take a different role, potentially acting as a common collaborating or general *trans*-acting factor associated with LDTFs (40, 43) at accessible chromatin in the hippocampus and other glucocorticoid-sensitive cell types.

### Relevance to disease

Our data suggest that factors that influence the expression, activity, and binding of NF-1/NeuroD1/Olig2 transcription factors might be expected to impact hippocampal GR binding and would be of interest in glucocorticoid-related disease. By way of example, seipin (*Bscl2*) knockout mice display a depressive phenotype accompanied by reduced *NeuroD1* expression (104), whereas mice exposed to 3 weeks of chronic mild stress undergo changes in *NeuroD1* expression prevented by the antidepressant agomelatine (105). Similarly, temporal lobe *Olig2* expression is downregulated in major depressive disorder (88) whereas antidepressant treatment preventing anhedonia in rodents upregulates *Nfib* in the frontal cortex following chronic mild stress (106, 107). It is possible that psychoactive drugs act in part by modification of accessible chromatin into which GRs bind, influencing the strength or type of glucocorticoid response. Equally, symptoms may arise from the impact of disease on transcription factors assisting GR loading. A dysregulated HPA axis might influence GR binding in this manner, as GR binding sites were found in and around genes for transcription factors linked to motifs underlying GR binding sites (Supplemental Fig. 3).

## Conclusions

We investigated the potential for stress context–dependent redistribution of GR binding in the hippocampus. We conclude that reorganization of GR binding sites in response to early stress mediators is not a mechanism for context-specific glucocorticoid actions in the rat hippocampus. Alternatively, however, it remains possible that chronic stress exposure might alter chromatin accessibility in a manner that could alter GR DNA binding. GR binding was found within or close to genes governing hippocampal structural organization, which is profoundly altered in neuropsychiatric disease. It remains to be seen whether such sites influence gene expression in acute or chronic stress paradigms. We additionally show that NF-1 is a key transcription factor associated with hippocampal GR binding. Despite displaying some properties expected of an LDTF, NF-1 half-sites are found within accessible chromatin from a variety of cell types, arguing against this role. NF-1 may instead serve as a collaborative or general transcription factor associated with GR function. bHLH transcription factors are extensively involved in hippocampal development with appropriate motifs found at GR binding sites. The motif obtained may relate to neurons or oligodendrocytes where previous data imply a lineage-determining role. Conditions that alter binding of NF-1 or NeuroD1/Olig2 may influence GR-mediated actions and have consequences for stress responses and disease susceptibility.
