# Supplementary material for: Genome-Wide Identification of Basic Helix–Loop–Helix and NF-1 Motifs Underlying GR Binding Sites in Male Rat Hippocampus
Source: Endocrinology. 2017 Feb 13;158(5):1486–501. doi: 10.1210/en.2016-1929 (PMC5460825; doi:10.1210/en.2016-1929)
Supplement: Supplementary file 6 [file en.2016-1929.sf1.pdf]

(a) Additional candidate motifs (full palindromic GRE containing peaks)

		p value	score (%)	% targets
Lhx3-like	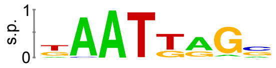	p=1e-31	90	17.0
Atoh1	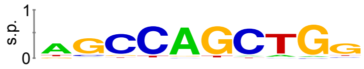	p=1e-27	84	32.0
AP-1 (Jun)	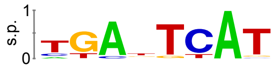	p=1e-20	89	7.8

(b) Additional candidate motifs (full palindromic GRE absent peaks)

		p value	score (%)	% targets
Egr2	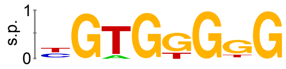	p=1e-29	83	28.4
Mef2a<br>Mef2c	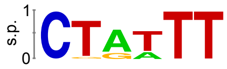	p=1e-22	85 (Mef2a)<br>84 (Mef2c)	32.8
