# Supplementary figures and images for: Genome-Wide Identification of Basic Helix–Loop–Helix and NF-1 Motifs Underlying GR Binding Sites in Male Rat Hippocampus
Source: Endocrinology. 2017 Feb 13;158(5):1486–501. doi: 10.1210/en.2016-1929 (PMC5460825; doi:10.1210/en.2016-1929)

### Distribution of Conventional and Negative GRE Motifs within Sites Containing nGREs

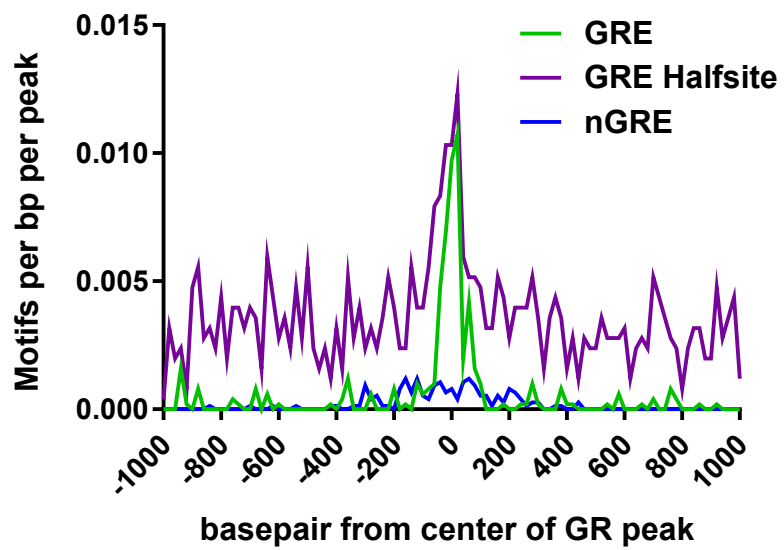

Supplement: Supplementary file 7 [file en.2016-1929.sf2.pdf]

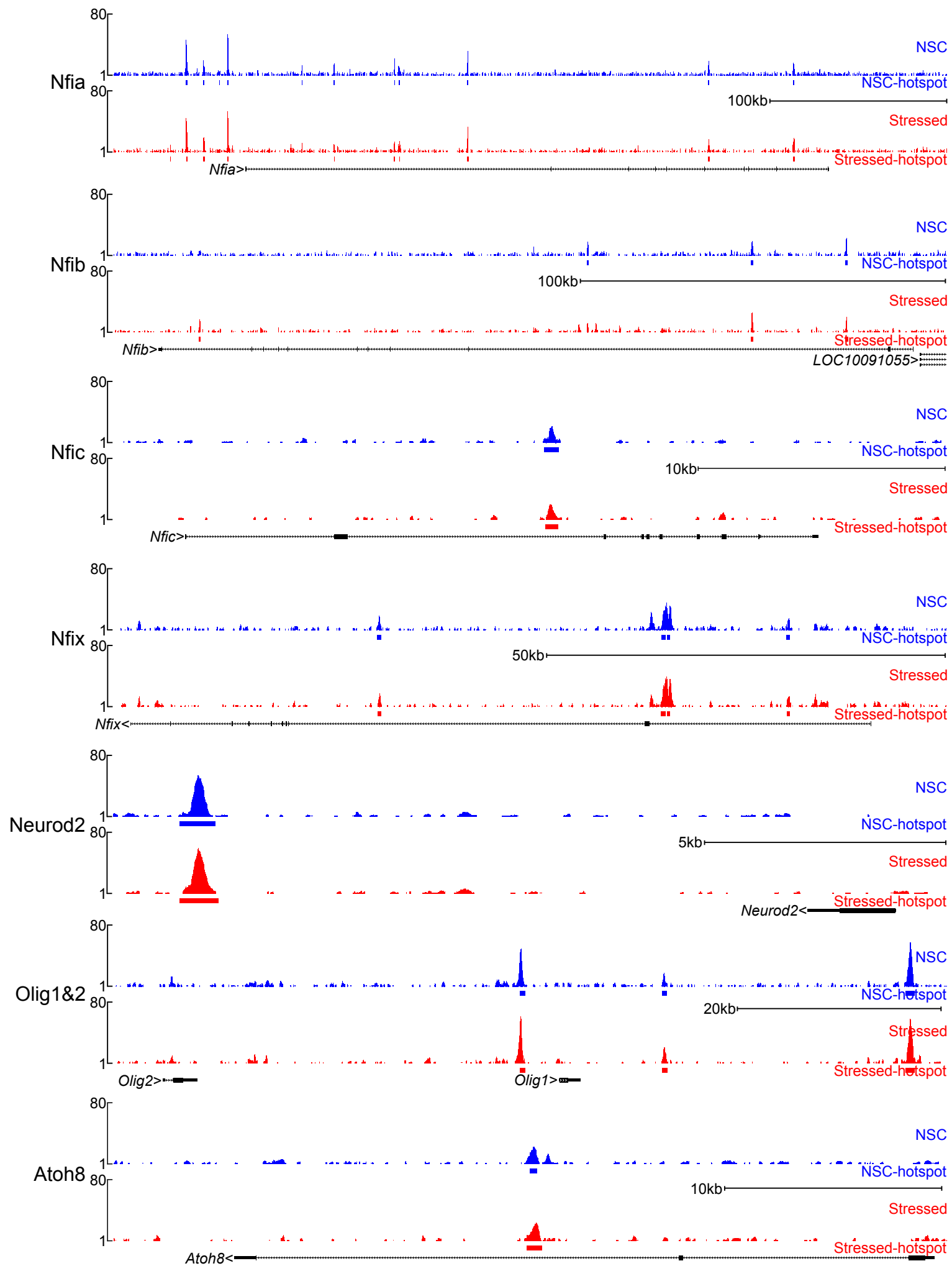

Supplement: Supplementary file 8 [file en.2016-1929.sf3.pdf]
